# Assessment of expression levels of leptin and leptin receptor as potential biomarkers for risk of prostate cancer development and aggressiveness

**DOI:** 10.1002/cam4.3082

**Published:** 2020-06-23

**Authors:** Hala Fawzy Mohamed Kamel, Anmar M. Nassir, Abeer A. Al refai

**Affiliations:** ^1^ Faculty of Medicine Biochemistry Department Umm Al‐Qura University Makkah Saudi Arabia; ^2^ Faculty of Medicine Medical Biochemistry Department Ain Shams University Cairo Egypt; ^3^ Urology Department Faculty of Medicine Umm Al‐Qura University Makkah Saudi Arabia; ^4^ Faculty of Medicine Medical Biochemistry and Molecular Biology Department Menoufia University Shebin Al‐Kom Egypt

**Keywords:** leptin, leptin receptor, prostate cancer

## Abstract

**Background:**

Prostate cancer (PCa) is one of the most frequently diagnosed cancers worldwide. Despite the growing evidence associating obesity and adipokines, particularly leptin and its receptors, with cancer development and progression, it is still a debatable matter in PCa.

**Objectives:**

We aimed to assess the role of leptin and its receptors as potential biomarkers for the risk of PCa development and aggressiveness.

**Methods:**

In this study, 176 men were included and categorized according to an established histopathological diagnosis into three age‐ and BMI‐matched groups. The PCa group included 56 patients while the BPH group and the control group comprised 60 men each. Serum levels of total PSA (tPSA) were assessed by ELISA and mRNA expression levels of leptin and leptin receptors were assessed by RT‐PCR.

**Results:**

Leptin and leptin receptor mRNA expression levels were significantly higher in PCa patients relative to BPH and to healthy control men. Both were overexpressed in PCa patients with aggressive and distantly metastasizing tumors compared to patients with confined tumors. Leptin receptor mRNA was an independent predictor of high Gleason score ≥ 7, distant metastasis, LN, and seminal vesicles invasion.

**Conclusion:**

Leptin and its receptors are suggested to be potential biomarkers for PCa; leptin receptor mRNA might predict risk and aggressiveness of PCa.

## Graphical abstract

This study revealed that the expression levels of leptin and leptin receptor mRNA are suggested to be the potential biomarkers for risk of prostate cancer. Leptin receptor mRNA expression might be considered an independent predictor of aggressive prostate cancer; further studies on larger cohort are needed to confirm these findings.

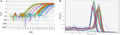

## 1 INTRODUCTION

Prostate cancer (PCa) has been reported to be one of the most frequently diagnosed cancers; it accounts for 7.1% of new cases of all cancers and 3.8% of cancer‐related deaths worldwide.^1^ Risks and associated factors of PCa include age, high androgen levels, overweight, and obesity.^2^ In fact, obesity has globally increased to an epidemic level and has been recognized as major health concern; aside from being related to diabetes, hypertension, and cardiovascular disease, it is linked to the development and progression of numerous types of cancers such as ovarian, pancreatic, endometrial, colon, postmenopausal breast, and prostate.^3^, ^4^, ^5^ Additionally, obesity has been associated with aggressive cancer, bad prognosis, poor outcome, and poor survival rate of cancer patients.^6^, ^7^, ^8^ Pathophysiological mechanisms underlying such association have not been fully elucidated; however, it may be related to insulin resistance, chronic inflammation, adipokines, and sex hormones dysfunctions.^9^ Expanded adipose tissue in obese subjects with release of adipokines are believed to interact with tumor microenvironment in several ways that could promote angiogenesis, invasion, and metastasis.^10^ Increased serum levels of adipocytokines, such as leptin, resistin, visfatin, and omentin, were associated with the risk and progression of several obesity‐interrelated cancers,^11^ we have reported that leptin and relatives may provide a link and potentially promising biomarkers for postmenopausal breast cancer.^12^, ^13^ Leptin is the most abundant and well‐characterized adipokine, it is secreted by adipocytes with a fluctuating level directly proportional to visceral fat accumulation and adipocyte mass in a direct proportion. Obese individuals have higher serum levels of leptin than normal or lean individuals.^14^, ^15^ Circulating serum levels of leptin were found to be significantly higher in advanced and high‐grade prostate cancer patients.^16^, ^17^

Leptin is a 16‐kDa protein that controls satiety, energy expenditure, and body weight.^18^ Leptin transmembrane receptors (Ob‐R) have about six isoforms; the long leptin receptor (Ob‐Rb) is the most predominant. Binding of leptin stimulates a cascade of signal‐transducing cascades of Janus kinase (JAK) and mitogen‐activated protein kinase (MAPK).^19^ In vitro studies have documented the mitogenic role of leptin in prostate cancer cell lines.^20^, ^21^, ^22^ Leptin promotes cell proliferation via MAPK stimulation^23^ and inhibition of apoptotic mechanisms in PCa cell probably via involvement of PI3K/Akt and MAPK.^24^, ^25^ In addition, leptin promotes proliferation of endothelial cells in androgen‐resistant cell lines of PCa (DU145 and PC‐3),^26^ which highlights the crucial role of leptin in induction of angiogenesis, progression, and metastasis in PCa. Thus, the long‐term exposure to elevated leptin levels, as in obese subjects, is a probable cause for increasing PCa risk or/and PCa aggressiveness.^27^ High expression levels of leptin and Ob‐R (class I cytokine‑receptor) have been observed in prostate tumors; leptin was found to be significantly higher in PCa than in benign prostatic hyperplasia (BPH) tissue.^28^ The short soluble isoform receptors (sOb‐Re) is circulating in serum as a carrier protein for leptin, binding occurs at 1:1 ratio and is involved in modulation of leptin's activity and bioavailability.^29^, ^30^ The sOb‐Re are proposed to delay leptin clearance from the circulation; additionally, they inhibit binding of leptin with their membrane receptors (Ob‐R).^31^ Leptin seems to modify the expression level of its sOb‐Re in opposing manner.^32^ In fact, the variation of sOb‐Re expression levels reflects similar changes in tissue or cellular receptors expression levels, similarly, the expression of Ob‐R is sensitive to experimentally induced changes in circulating leptin.^33^

Leptin exerts its effects through the interaction with the specific Ob‐R via signal transduction cascade.^34^ Moreover, it seems to be involved in exerting mitogenic and proliferative effects.^34^ Thus, dysregulation of signaling pathways of leptin and their receptors might be involved in the emergence of cancer.^35^

Despite the growing evidence linking obesity and adipokines namely leptin and its receptors with PCa, it is still a matter of debate.^17^, ^36^, ^37^ Therefore, our objectives were to assess the levels of expression of leptin and leptin receptors mRNA in PCa patient, BPH, and healthy subjects in order to evaluate their role as potential biomarkers for risk of PCa development and aggressiveness.

## 2 METHODS

Our study population included 176 men; all of which were enrolled during the period of January 2016 to December 2018. All participants were informed of the aim of our study. They signed a consent form agreeing to participate in this study while maintaining their anonymity in complying with The Code of Ethics of the World Medical Association (Declaration of Helsinki). The included subjects were categorized into three age‐ and BMI‐ matched groups according to the established diagnosis for each group. The first group is PCa group consisting of 56 histopathologically confirmed male patients’. The second group is the BPH group consisting of 60 males who were confirmed by histopathological examination of the transurethral resection biopsies (TURPs) to have benign hyperplasia of prostate. The last group is the control group consisting of 60 apparently healthy men who were recruited from attending males to outpatient clinic of the same ethnicity of patients. Exclusion criteria for our cohort weres cases with acute urinary tract infection, acute or chronic prostatitis, patients with past history of any urogenital cancers, and chronic kidney disease. All subjects were subjected to full history taking, general, and local clinical examinations. Prostate cancer patients with confirmed diagnosis by histopathology were recruited from Urology department; blood samples were obtained prior to any surgical, hormonal, radiotherapy intervention, and/or any other therapy modalities. Therefore, any influence of treatment was unlikely. BMI was calculated as weight in kilogram divided by height in square meters. Detailed clinicopathological and surgical data for the PCa group included but was not limited to TNM stage, Gleason score, LN, histological grade safety margin, and seminal vesicles.

From each patient, 10 mL of venous blood were collected in two tubes: vacutainer plain tube for assessment of serum total PSA (tPSA) and vacutainer EDTA‐containing tube for detection of leptin and leptin receptor mRNA expression levels in the blood samples. Serum tPSA was assessed using enzyme‐linked immunosorbent assay (ELISA) technique and kit for measurement of tPSA is brought from Sunlong Biotech Co. Ltd, Zhejiang, China; (Cat NO.:SL1727Hu). Blood samples were evaluated blindly to the diagnostic information in duplicate according to the manufacturer's instructions with all the quality control measurements within the ranges recommended by the manufacturers.

Measurement of leptin and of leptin receptor mRNA expression levels in blood samples was performed using reverse transcriptase PCR (RT‐PCR) by means of real‐time PCR, RNA was extraction from peripheral blood leukocytes using a QIAamp RNA Blood Mini Kit (Qiagen, 2013), then assuring RNA concentration and purity by Nano drop. The purified RNA was stored at −70°C until further steps and procedures. In the first‐step PCR, complementary DNA (cDNA) was synthesized using a QuantiTect Reverse Transcription Kit (Qiagen, Applied Biosystems, 2012), In the second–step PCR (real‐time PCR step), it was performed using a QuantiTect SYBR Green PCR Kit with a readymade quantiTect Primer Assay,^38^ Qiagen. For measurement of leptin and leptin receptor mRNA levels, the following primers were used: forward and reverse primers of human leptin (NM 000230.2), 5‐TCCCCTCTTGACCCATCTC‐3 and 5‐GGGAACCTTGTTCTGGTCAT‐3, respectively; forward and reverse primers for human leptin receptor (NM001003679.2) 5‐AGGAAGCCCGAAGTTGTGTT‐3 and 5‐TCTGGTCCCGTCAATCTGA‐3, respectively. In addition, beta‐2‐microglobulin was used as a control gene using the following forward 5‐CTATCCAGCGTACTCCAAAG‐3 and reverse primers 5‐ACAAGTCTGAATGCTCCACT‐3 were utilized, respectively. PCR was conducted on Applied Biosystem 7500 RT‐PCR under the following conditions: 40 cycles; denaturation at 95°C for 15 seconds, annealing at < 60°C for 15 seconds, and extension at 72°C for 60 seconds. (Standard cycling conditions are recommended). Our results were analysed by calculating the baseline and threshold cycles (CT) for the amplification curves using software version: 2.0.1 and perform relative and absolute quantitation.

## 3 RESULTS

Participants in this study included 56 males with PCa, 60 with BPH, and 60 apparently healthy individuals. The average age of patients in the BPH, PCa and control groups was 59.7 ± 3, 60.3 ± 2.1, and 59.4 ± 2.7 years, respectively, which showed no statistical difference (*P* = .19). In the terms of BMI, it was 28.8 ± 2.5, 28.8 ± 2.2, and 27.9 ± 2 for the BPH, PCa and control groups, respectively, revealing a nondistinctive difference (*P* = .08); also, the studied groups categorized into lean, overweight, and obese subgroups based on BMI showed nonsignificant difference (*P* = .17). Baseline characteristics, and leptin and leptin receptor mRNA expression levels in the blood are shown in Table 1. Post hoc test of one‐way ANOVA for the studied groups categorized by BMI revealed significant upregulation of leptin mRNA expression among the PCa subgroups stratified by BMI compared to all other BPH and control subgroups (*P* = .0001). Meanwhile, leptin receptors mRNA expression levels exhibited significant difference between overweight PCa and all other subgroups; similarly significant difference was found in obese PCa in comparison to the other subgroups except for lean PCa and obese control (*P* = .0001).

**Table 1:** Baseline characteristics for cases and referents

Variables	PCa 56	BPH 60	Control 60	*P* value
Age (X ± SD)	60.3 ± 2.1	59.7 ± 3	59.4 ± 2.7	.19
BMI (X ± SD)	28.8 ± 2.2	28.8 ± 2.5	27.9 ± 2.4	.08
BMI category				
Lean N (%)	11 (19.6%)	8 (13.3%)	13 (21.7%)	
Overweight N (%)	31 (55.5%)	33 (55%)	39 (65%)	.17
Obese N (%)	14 (25%)	19(31.7%)	8 (13.3%)	
tPSA (X ± SD)	11.01 ± 3.02	4.03 ± 2.02	2.9 ± 1.15	.0001*
Leptin mRNA Median (Range)	12.1 (85)	0.1 (6)	0.03 (0.98)	P1 = .0001* P2 = .06 P3 = .0001*
Leptin mRNA Category				
Lean	12.3 (0.3)	0.01 (0.01)	0.024 (0.98)	
Overweight	85 (81)	0.1 (6)	0.025 (0.98)	.0001*
Obese	7.4 (4.4)	0.23 (2.22)	1 (0.98)	
Leptin R mRNA Median (Range)	1.6 (5.96)	0.13 (1.98)	0.3 (4.9)	P1 = .0001* P2 = .39 P3 = .0001*
Leptin mRNA category				
Lean	1.17 (0.1)	0.1 (0.05)	0.1 (0.9)	
Overweight	5.1 (5.96)	0.13 (1.98)	0.1 (1.1)	.0001*
Obese	3.9 (3.96)	1 (1.5)	0.5 (4.5)	
Clinical stage		–	–	
Early (cT1; cT2a) N (%)	22 (39.3%)			
Late (cT2b, c; T3) N (%)	34 (60.7%)			
Gleason score		–	–	
<7 N (%)	28 (50%)			
≥7 N (%)	28 (50%)			
Surgical margin				
−ve N (%)	29 (51.8%)			
+ve N (%)	27 (48.2%)			
Lymph N		–	–	
−ve N (%)	31 (55.4%)			
+ve N (%)	25 (44.6%)			
Seminal V Invasion		–	–	
−ve N (%)	31 (55.4%)			
+ve N (%)	25 (44.6%)			
Metastasis		—	–	
−ve N (%)	43 (76.8%)			
+ve N (%)	13 (23.2%)			

Note

P1: PCa vs BPH; P2: BPH vs Control; P3: PCa vs Control.

* Post hoc test revealed significant difference among studied groups.

### 3.1 Leptin and leptin receptor expression levels in the three studied groups

**Figure 1:**
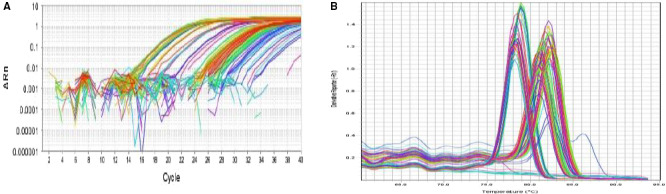
Reference gene and target gene expression analysis by the SYBR Green qPCR method for both leptin and leptin receptor genes (A) the amplification plot of the analyzed genes expression [Log ΔRn vs cycles, the plot colors represent the corresponding wells] (B) melting curve of the expressed genes [−Rn vs temperature]

Leptin and leptin receptor relative expression levels was performed between the BPH, PCa, and control groups by RT‐PCR (Figure 1). The expression levels of leptin and leptin receptor mRNA was upregulated in the PCa group compared to both the BPH and control groups (*P* = .0001). However, the expression of leptin and leptin receptor exhibited nonsignificant difference between the BPH and control groups (*P* = .06 and 0.39), respectively.

### 3.2 Leptin and leptin receptor mRNA expression in different clinicopathological features of PCa cases

We classified PCa patients according to the Gleason score into two subgroups, the PCa group with a high Gleason score (≥7) had higher leptin and leptin receptor expression levels than the low Gleason score patients (<7) (*P* = .02 and 0.002), respectively (Table 3). The patients with metastatic PCa exhibited higher values of leptin and leptin receptor mRNA expression compared to that of the localized PCa patients (*P* = .0001). Similarly, those with lymph node and seminal vesicle invasion (*P* = .007, 0.005, and 0.01), respectively, Table 2.

**Table 2:** Leptin and leptin receptor mRNA expression and clinicopathological features of PCa cases

	Leptin mRNA median (Range)	*P* value	Leptin receptor mRNA median (Range)	*P* value
Gleason score				
˂7 (28)	12 (82)	.02	1.17 (5.9)	.002
≥7 (28)	28.6 (81)		4 (5.1)	
Clinical stage				
Early (cT1;cT2a) (22)	12.15 (82)	.45	1.17 (5.9)	.03
Late (cT2b,c;T3) (34)	12.1 (85)		3.97 (5.1)	
Surgical margin				
Negative (29)	12 (82)		1.2 (5.96)	
Positive (27)	45 (81)	.01	4 (5.1)	.003
Seminal vesicles invasion				
Negative (31)	12 (82)	.007	1.2 (5.9)	.005
Positive (25)	88 (81)		5 (5.1)	
Lymph node invasion				
Negative (31)	12 (82)	.01	1.2 (5.9)	.01
Positive (25)	88 (81)		5 (5.1)	
Distant metastasis				
Negative (43)	12 (85)	.0001	1.2 (5.9)	.0001
Positive (13)	88 (80.6)		5.14 (1.14)	

### 3.3 Correlation between leptin and leptin receptor mRNA expression and studied parameters among BPH and PCa patients

Among BPH and PCa patients, the results demonstrated a significant positive correlation between leptin mRNA expression and leptin Receptor mRNA expression (*P* = .0001), serum tPSA (*P* = .011), BMI (*P* = .001), and age (*P* = .0001). In the same manner, there was also significant positive correlation between leptin receptor mRNA expression and these variables except for age Tables 3 and 4.

**Table 3:** Correlation between leptin and leptin receptor mRNA expression and studied parameters among BPH

Studied parameters	BPH (N = 60)			
	Leptin mRNA		Leptin R mRNA	
	*r*	*P*	*r*	*P*
Age	0.02	.8	−0.17	.17
BMI	0.3	.01	0.36	.004
Leptin mRNA	—	—	0.44	.0001
Leptin receptor mRNA	0.44	.0001	—	—
tPSA	0.3	.02	0.18	.16

**Table 4:** Correlation between leptin and leptin receptor mRNA expression and studied parameters among PCa

Studied parameters	Studied parameters			
	Leptin mRNA		Leptin R mRNA	
	*r*	*P*	*r*	*P*
Age	0.55	.0001	0.15	.24
BMI	0.4	.001	0.15	.25
Leptin mRNA	—	—	0.7	.0001
Leptin receptor mRNA	0.7	.0001	—	—
tPSA	0.3	.011	0.28	.034

### 3.4 Leptin and leptin receptor mRNA expression as a risk for PCa and PCa aggressiveness

Linear regression analysis was performed to explore the relative risk of PCa. In linear regression analysis, the relative risk of prostate cancer was associated only with serum tPSA and leptin receptor mRNA expression (*P* = .0001). Leptin mRNA showed negative association with risk of prostate cancer (*β* = −0.23); however, it did not reach a significant level (*P* = .063,). This negative association could be attributed to the availability of other variables such as leptin receptor mRNA and tPSA (Table 5).

**Table 5:** Linear regression analysis for independent variables of PCa

Variables	PCa		
	*β*	*P*	CI
Age	0.1	.09	−0.4‐0.04
BMI	−0.07	.2	−0.04‐0.009
tPSA	0.67	.0001	0.06‐0.09
Leptin mRNA	−0.23	.063	−0.008‐0.000
Leptin R mRNA	0.43	.0001	0.5‐0.17

Furthermore, the relative risk of PCa aggressiveness (Gleason score (≥7), seminal V invasion, and LN invasion) was associated with serum tPSA (*P* = .0001), leptin receptor mRNA expression (*P* = .042, 0.043, and 0.04), and demographic data. In contrast, linear regression analysis revealed that relative risk of distant metastasis was associated with serum tPSA (*P* = .0001) and leptin mRNA expression (*P* = .004) Table 6.

**Table 6:** Linear regression analysis for independent variables of aggressiveness in PC

Variables	*β*	*P*	CI
Gleason score			
tPSA	0.54	.0001	0.05‐0.13
Leptin R mRNA	0.38	.042	0.003‐0.18
L N invasion			
Age	0.44	.034	0.008‐0.2
BMI	0.28	.012	0.015‐0.12
tPSA	0.56	.0001	0.05‐0.12
Leptin R mRNA	0.64	.043	0.005‐0.3
Seminal V invasion			
Age	0.45	.03	0.01‐0.2
BMI	0.3	.01	0.02‐0.12
tPSA	0.6	.0001	0.05‐0.12
Leptin R mRNA	0.6	.04	0.01‐0.3
Distant metastasis			
tPSA	0.5	.0001	0.05‐0.09
Leptin mRNA	0.7	.004	0.003‐0.014

## 4 DISCUSSION

In this study, PCa patients had significantly higher leptin and leptin receptor mRNA expression levels relative to the BPH and control groups. Both leptin and its receptors showed greater expression levels in the BPH than in the control group, but it was not statistically significant. Numerous studies and epidemiological data have indicated the role of adipokines (particularly leptin) in the development and the progression of cancer. Enhanced expression of leptin and leptin receptors has been reported in breast cancer in comparison to normal breast tissue and was significantly related to distant metastasis.^39^ Also, the overexpression of leptin and leptin receptors was related to the development and the degree of gastric cancer,^40^ and endometrial cancer.^41^ Overexpression of leptin receptors and elevated leptin levels in the serum is cardinally related to tissue invasion of renal cell carcinoma.^42^ Similarly in prostate cancer; Kim et al. observed higher expression of leptin in PCa than in BPH specimens by immunohistochemical technique; however, they did not find significant difference between BPH and normal tissue. They reported that leptin might stimulate occurrence and progression of PCa.^28^ Likewise, immunoreactive leptin receptors were observed in high‐grade precancerous PIN lesions as well as in prostatic cancer tissue, while no immunoreaction was detected in normal prostatic stroma.^43^ Serum leptin levels were significantly different in PCa vs healthy control and inpatients with benign prostatic lesions, levels were related to tPSA and associated with testosterone in PCa patients.^44^ However, *Lagiou et al* could not find a significant relation between serum levels of leptin with either the development of BPH or the progress of PCa in elderly men.^45^ In vitro studies suggested the role of leptin and its receptors in the development of prostate cancer. Nevertheless, the exact pathogenic mechanism remains inconclusive. Leptin was reported to induce cell proliferation in PC‐3 and DU145 human PCa cell lines through JNK activation cascade.^22^ Moreover, it enhances the progression and survival of PCa cells via PI3K/Akt or ERK1/2 pathways according to the type of cells.^46^ In a dose‐dependent manner, leptin induces proliferation, mitogenic, and antiapoptotic effects when it is cultured with human PCa cell lines, revealing that chronic exposure to high‐leptin levels as in obesity could promote the progression of PCa via the MAPK and PI3K signaling cascade.^24^

Lately, several reports found an empirical connection between obesity (namely leptin or its receptors) with several types of cancer.^5^, ^47^, ^48^ However, the studies which investigated the association between obesity and PCa risk are debatable and inconclusive.^49^ Our results revealed that leptin receptor mRNA expression in the blood was an independent predictor and was associated with increased risk of PCa. Some reports agreed with these findings, they found a positive correlation between PCa risk and serum leptin or leptin receptor expression in prostate tissue.^17^, ^28^, ^43^ However, the range of obesity does not influence the values of leptin expression in PCa tissue, unlike the leptin values in blood.^28^

*Hsing et al* hypothesized that leptin intermingles with insulin, sex hormones, and growth factors such as IGF‐1 in patients with abdominal obesity and high waist to hip ratio (≥ 0.87) which might increase PCa risk.^36^ In contrast, others concluded that there were no association between serum leptin and risk of PCa.^37^, ^45^ There were numerous reports investigating the association of leptin or leptin receptors mRNA expression with the risk of various cancers either by immunohistochemical techniques or by RT‐PCR (as this study); however, fewer studies have been conducted on prostate cancer.

Enhanced expression of leptin and its receptors has a positive association with the risk of breast,^39^, ^50^ endometrial,^41^, ^51^ colorectal,^52^ gastric,^35^, ^40^ ovarian,^53^ and upper tract urothelial carcinomas.^54^ For PCa risk, Kim and his colleagues found a strong positive association with leptin expression; however, they found no correlation between leptin receptor expression values and PCa occurrence.^28^ Their cohort study groups included only PCa and BPH but not normal prostate tissue unlike our studied groups. Additionally, their sample size was smaller than ours and they assessed leptin and leptin receptor expression levels in tissue using immunohistochemistry semiquantitative scale while we assessed expression levels of leptin and its receptors mRNA in blood samples using RT‐PCR. In agreement with our findings, they concluded that the association between leptin and PCa risk was BMI independent. In fact, BMI is not a perfect reliable indicator or a surrogate marker for fat mass or adiposity because it has some limitations such as site of obesity weather abdominal, hip, or all over the body. Also, composition of body, muscular and thick‐bony built, are not considered in calculation formula of BMI; therefore, other markers for body fat or adiposity have been used in risk assessment studies like anthropometric measures such as waist circumference, waist to hip ratio; percentage of body fat, crude weight, and body mass.^55^

A previous retrospective study among a cohort of 135 006 Swedish men, who were under follow‐up for around 20 years, concluded that anthropometric measures of obesity as BMI and lean body mass possessed a stronger association with the risk of death from advanced or fatal PCa rather than the risk of occurrence of PCa.^56^ Likewise, BMI was reported to be inversely correlated to confined and lower grade PCa, yet it was positively associated with the risk of metastatic PCa.^57^ Substantially, a robust association of BMI with PCa aggressiveness was found while no association of BMI was encountered with an overall risk for PCa in a meta‐analysis study.^49^ In the same context, obesity might have stronger association with PCa aggressiveness than PCa incidence.

In our studied PCa group, levels of leptin and leptin receptor mRNA expression were significantly higher in patients with aggressive PCa (Gleason score ≥ 7, late stage, LN, seminal vesicles or surgical margin invasion and distantly metastasizing tumors) in comparison to PCa patients with confined and less aggressive tumors. We found that leptin receptor mRNA was an independent predictor of Gleason score ≥ 7, distant metastasis, LN, and seminal vesicles invasion. Furthermore, expression levels of leptin mRNA and its receptors were positively associated with tPSA, suggesting that leptin might be a biomarker of PCa aggressiveness. These findings point to the crucial role of leptin and its receptors in promoting the progression of PCa and suggest the potential value of leptin receptor for prediction of adverse outcomes. In agreement with our results, leptin expression was significantly higher in PCa specimens with Gleason scores 8‐10 compared to the Gleason scores ≤ 6; metastasizing tumors showed higher expression levels relative to locally advanced tumors and localized tumors.^28^ Likewise, in other reports, serum leptin levels were observed to be significantly higher in advanced prostate cancer relative to confined tumors.^58^ Increase in leptin might be closely linked to the occurrence and progression of PCa.^59^

Lopez and his colleagues reported that serum leptin levels were significantly higher in PCa patients with a high Gleason score.^60^ In several studies, leptin was positively correlated to aggressiveness, advanced histological grade, or stage of PCa.^16^, ^17^, ^60^, ^61^ Larger volume prostate tumors and higher histological grade PCa have been associated with significantly higher serum leptin levels than less volume or less advanced tumors.^16^, ^17^ Hence, leptin may be more valuable in prediction of aggressive PCa than in prediction of development of PCa. However, further confirmatory studies are needed. On the molecular level, leptin induces proliferation of endothelium in vivo and in vitro, via upregulation of vascular endothelial growth factor (VEGF) and induction of matrix metalloproteinases (MMP); these effects have crucial role in mediating LN invasion and distant metastasis.^62^ Leptin enhances PCa cell migration by stimulating expression of growth factors such as transforming growth factor‐beta1 (TGF‐beta1) and basic fibroblast growth factor (b‐FGF). Such effects were inhibited by adding MAPK and PI3K inhibitors indicating the critical role of leptin in PCa progression and aggressiveness.^26^ Leptin induction of prostate cell proliferation was proposed to be through reciprocal effect of leptin on estrogen metabolism. Leptin induces the expression of estrogen receptor (ER)‐α and represses ER‐β expression as well.^63^

## 5 CONCLUSIONS

In conclusion, this study revealed that expression levels of leptin and leptin receptor mRNA are suggested to be potential biomarkers for PCa. Additionally, leptin receptor mRNA expression might be considered an independent predictor of risk and aggressiveness of PCa; however, further studies on larger cohort are needed to confirm these findings.

## CONFLICT OF INTEREST

The authors declare that they have no conflict of interests.

## AUTHOR CONTRIBUTIONS

Conceptualization: Anmar M. Nassir and Hala FM Kamel; Formal analysis: Hala FM Kamel and Abeer A. Al refai; Funding acquisition: Anmar M. Nassir; Investigation: Hala FM Kamel and Abeer A. Al refai; Resources: Anmar M. Nassir; Writing—original draft: Hala FM Kamel; Writing—review and editing: Anmar M. Nassir, Hala FM Kamel, and Abeer A. Al refai.

## ETHICAL APPROVAL

The Ethics Review Board for Human Studies of Faculty of Medicine, Umm Al‐Qura University approved this study; protocol number was (HAPO‐02‐K‐012‐2015‐01‐103).
